# Supplementary material for: A generalizable nanopore sensor for highly specific protein detection at single-molecule precision
Source: Nat Commun. 2023 Mar 20;14:1374. doi: 10.1038/s41467-023-36944-9 (PMC10027671; doi:10.1038/s41467-023-36944-9)
Supplement: Supplementary file 2 — Reporting Summary [file 41467_2023_36944_MOESM2_ESM.pdf]

nature portfolio

Corresponding author(s): Liviu Movileanu

Last updated by author(s): Feb 4, 2023

Reporting Summary

Nature Portfolio wishes to improve the reproducibility of the work that we publish. This form provides structure for consistency and transparency in reporting. For further information on Nature Portfolio policies, see our Editorial Policies and the Editorial Policy Checklist.

Statistics

For all statistical analyses, confirm that the following items are present in the figure legend, table legend, main text, or Methods section.

n/a	Confirmed	
☐	☒	The exact sample size (*n*) for each experimental group/condition, given as a discrete number and unit of measurement
☐	☒	A statement on whether measurements were taken from distinct samples or whether the same sample was measured repeatedly
☐	☒	The statistical test(s) used AND whether they are one- or two-sided<br>*Only common tests should be described solely by name; describe more complex techniques in the Methods section.*
☒	☐	A description of all covariates tested
☒	☐	A description of any assumptions or corrections, such as tests of normality and adjustment for multiple comparisons
☐	☒	A full description of the statistical parameters including central tendency (e.g. means) or other basic estimates (e.g. regression coefficient) AND variation (e.g. standard deviation) or associated estimates of uncertainty (e.g. confidence intervals)
☒	☐	For null hypothesis testing, the test statistic (e.g. *F*, *t*, *r*) with confidence intervals, effect sizes, degrees of freedom and *P* value noted<br>*Give P values as exact values whenever suitable.*
☒	☐	For Bayesian analysis, information on the choice of priors and Markov chain Monte Carlo settings
☒	☐	For hierarchical and complex designs, identification of the appropriate level for tests and full reporting of outcomes
☒	☐	Estimates of effect sizes (e.g. Cohen's *d*, Pearson's *r*), indicating how they were calculated

Our web collection on statistics for biologists contains articles on many of the points above.

Software and code

Policy information about availability of computer code

Data collection	pClamp 10.7, Clampex 10.7, FortéBio Octet HT aquisition 11, and SoftMax Pro 6.4
Data analysis	pClamp 10.7, ClamFit 10.7, Origin 9.7, MATLAB R2021a, FortéBio Octet HT kinetic analysis 11, PyMOL 2.4, Chimera X 1.4, and AlphaFold2. All custom codes and mathematical algorithms are available at https://doi.org/10.5281/zenodo.7504671.

For manuscripts utilizing custom algorithms or software that are central to the research but not yet described in published literature, software must be made available to editors and reviewers. We strongly encourage code deposition in a community repository (e.g. GitHub). See the Nature Portfolio guidelines for submitting code & software for further information.

Data

Policy information about availability of data

All manuscripts must include a data availability statement. This statement should provide the following information, where applicable:

- Accession codes, unique identifiers, or web links for publicly available datasets
- A description of any restrictions on data availability
- For clinical datasets or third party data, please ensure that the statement adheres to our policy

In addition to the Supplementary Information file, data supporting the findings of this article have been deposited in the Zenodo database at https://doi.org/10.5281/zenodo.7504671. The source data underlying Figs. 2b-g, 3b-g, 4b-f, 5d-k, and Supplementary Figs. 6-7, 14b, 24-25 and Supplementary Tables 2-20 are provided in the Source data file. Entries 3RZW [http://doi.org/10.2210/pdb3RZW/pdb], 6BYN [http://doi.org/10.2210/pdb6BYN/pdb], 1NQL [http://doi.org/10.2210/pdb1NQL/pdb], 3QWQ [http://doi.org/10.2210/pdb3QWQ/pdb], 1BY3 [http://doi.org/10.2210/pdb1BY3/pdb], and 1FNF [http://doi.org/10.2210/

pdb1FNF/pdb] from Protein Data Bank were used in this article for visualizations and molecular graphics. Source data are provided with this paper.

Human research participants

Policy information about studies involving human research participants and Sex and Gender in Research.

Reporting on sex and gender	None
Population characteristics	None
Recruitment	None
Ethics oversight	None

Note that full information on the approval of the study protocol must also be provided in the manuscript.

Field-specific reporting

Please select the one below that is the best fit for your research. If you are not sure, read the appropriate sections before making your selection.

- [x] Life sciences
- [ ] Behavioural & social sciences
- [ ] Ecological, evolutionary & environmental sciences

For a reference copy of the document with all sections, see nature.com/documents/nr-reporting-summary-flat.pdf

Life sciences study design

All studies must disclose on these points even when the disclosure is negative.

Sample size	Single-channel traces were recorded for long periods, so the sample size encompassed several hundred events. This way, satisfactory reproducibility was achieved from one experiment to the next, as indicated by values of standard deviations.
Data exclusions	No data were excluded from this analysis.
Replication	Experiments were performed using samples from different protein preparations. At least three independent experiments were conducted under similar conditions on different days. All attempts at replication were successful.
Randomization	Randomization is not applicable to this study because no distinct treatments/interventions/procedures were applied to different groups of experiments/samples, such as those from clinical trials.
Blinding	Blinding was not relevant to this study because of the uniform data collection and statistical analysis throughout this work. Identical sample preparations and similar research protocols were required to warrant the main conclusions in this study. Again, no distinct treatments/interventions/procedures were applied to different groups of experiments/samples, such as those from clinical trials.

Reporting for specific materials, systems and methods

We require information from authors about some types of materials, experimental systems and methods used in many studies. Here, indicate whether each material, system or method listed is relevant to your study. If you are not sure if a list item applies to your research, read the appropriate section before selecting a response.

Materials & experimental systems

n/a	Involved in the study	
x		Antibodies
	x	Eukaryotic cell lines
x		Palaeontology and archaeology
x		Animals and other organisms
x		Clinical data
x		Dual use research of concern

Methods

n/a	Involved in the study	
x		ChIP-seq
x		Flow cytometry
x		MRI-based neuroimaging

Eukaryotic cell lines

Policy information about cell lines and Sex and Gender in Research

Cell line source(s)	Expi293F cells (Thermo Fischer Scientific; Cat# A14527)
Authentication	No authentication procedure was used.
Mycoplasma contamination	The cell line was not tested for mycoplasma contamination.
Commonly misidentified lines (See ICLAC register)	This is not applicable.
